# The role of vocational education in the transmission of gender segregation from education to employment: Switzerland and Bulgaria compared

**DOI:** 10.1186/s12651-018-0248-6

**Published:** 2018-12-11

**Authors:** Melina Heiniger, Christian Imdorf

**Affiliations:** 1 Institute of Sociology, University of Bern, Fabrikstrasse 8, 3012 Bern, Switzerland; 2 Department of Sociology and Political Science, Norwegian University of Science and Technology NTNU, Dragvoll, 7491 Trondheim, Norway; 3 Institute for the Study of Societies and Knowledge, Bulgarian Academy of Sciences, Ulitsa Serdika 4, 1000 Sofia, Bulgaria

**Keywords:** Gender segregation, Vocational education, Labour market, Education–employment linkage, Bulgaria, Switzerland, I21, J16, J21, P36, Z13

## Abstract

Previous comparative research has uncovered considerable cross-country differences in occupational gender segregation. There is, however, a lack of research on the role of educational systems in the creation of gender segregation and gendered school-to-work transitions. The aim of this study is to investigate the role of vocational education and the strength of the education–employment linkage in the transmission of horizontal gender segregation from education into the labour market. Transition system literature points to a stronger linkage between education and employment in countries where initial vocational education and training dominates the educational offers, and to a weaker linkage in countries with a stronger focus on general education. Moreover, research on gender segregation in education shows that segregation is especially pronounced in educational systems with a strong vocational education and training sector on the upper secondary level. Based on these insights, we hypothesize that gender segregation in education and its transmission to employment is more pronounced the more distinct a country’s initial vocational education and training system is. To test our assumption, we compare individual school-to-work transitions in Switzerland and Bulgaria, with the vocational principle being more prevalent in the structuring of Swiss educational offers. We use data from the Swiss Youth Panel Survey TREE (N = 3215) and the Bulgarian School Leaver Survey BSLS (N = 885). Following recent developments in multi-group segregation research, entropy-based measurements are calculated to study the school-to-work linkages and the transmission of gender segregation in the two select countries. The empirical results confirm a more pronounced educational gender segregation in Switzerland, which is transferred more strongly into the labour market due to the tighter linkage in that country between education and employment compared to Bulgaria.

## Introduction

The educational chances of women have improved in most western countries due to educational expansion, to the extent that now women hold higher educational levels compared to men in many places (DiPrete and Buchmann 2013). At the same time, the structure of the labour market has changed. The primary and secondary sectors of the economy (with many male-typed jobs in agriculture and industry) are shrinking in many countries, while the service sector is growing (Hakim 1996). The latter has also grown because traditionally female-typed domestic work like childcare has been outsourced to the labour market (Steinmetz 2012). Thus, the labour market chances of women have not only improved due to their raised human capital, but also because of structural shifts in the economy (Charles 2003).

Despite these factors, labour markets remain strongly horizontally segregated (Smyth and Steinmetz 2008). Although horizontal gender segregation (i.e. men and women working in different occupations) does not necessarily imply inequality between the genders, it can have problematic effects in countries where horizontal gender segregation is linked to vertical segregation. Where female-typed jobs are characterized by lower salaries, worse advancement prospects, fewer opportunities for further education, and worse working conditions, horizontal segregation can be an important cause of vertical inequality between men and women (Buchmann and Kriesi 2009). Furthermore (and relatedly), beside self-selection, women with female-typed occupational certificates may be pushed into ‘part-time friendly’ occupations and to accept the burdens of unpaid childcare and housework, through which the traditional division of labour within the job market and within the family is maintained and the male breadwinner model may persist (Levy 2013). Hence, horizontal gender segregation can be considered problematic with respect to unequal chances for men and women in the life course, and it therefore deserves a more comprehensive understanding.

The research literature presents different approaches that seek to explain occupational gender segregation. Studies grounded in socialization theory are prevalent, assuming that gender segregation is a result of gender stereotypical role models that become internalized in the process of socialization. In contrast, economic perspectives, such as human capital theory, conceptualize men and women as rational actors who choose the best profession for themselves based on a cost–benefit analysis. Here, gender segregation arises through gender-typical preferences and restrictions that affect such a cost–benefit analysis. Finally, there are institutional explanations according to which gender segregation is shaped by institutional contexts of education and work (Reisel et al. 2015).

The present study aims to investigate the under-researched institutional design of the educational system as an explanation for horizontal gender segregation. We are particularly interested in the linkage between education and employment, as well as in the role played by vocational education and training (VET) in the transmission of gender segregation from education into the labour market. Previous research has shown that educational systems with strong vocational orientation exhibit stronger horizontal gender segregation compared to general educational systems (Reisel et al. 2015). We assume that in a country-context with extended VET the linkage between education and employment is especially pronounced, and that the gender segregation in education is therefore transmitted to a greater extent into the labour market.

To test our assumptions, we compare individual transitions from education into employment in Switzerland and Bulgaria. We chose those two countries for conceptual as well as for pragmatic reasons. Those two countries differ in the extent to which horizontal gender segregation exists in the labour market; it is historically higher in Switzerland compared to Bulgaria, whereas they have similar shares of women in general upper secondary education and in higher education (Sect. 2.2). Furthermore, whereas both countries have a widely developed and highly gender segregated vocational education system, the vocational principle (*Berufsprinzip*) is more strongly pronounced in the post-obligatory educational system of Switzerland, where occupational skills development produces more specific qualifications that are more targeted to meet an employer’s needs. In contrast, mostly general knowledge is taught as a relevant occupational skill in the Bulgarian VET system. While, during the state socialist era, education served the goals of the centrally planned economy that corresponded to the various economic sectors, the relation with enterprises was eliminated and vocational education in Bulgaria became increasingly school-based after 1989 (Ilieva-Trichkova et al. 2015; Rokicka et al. 2018). Hence, the education–employment linkage can be assumed to be stronger in Switzerland than in Bulgaria, even though an extensive VET system prevails in both countries.

Based on the outlined differences, the two countries are well-suited for a comparative investigation of the effect of the institutional design of the educational system on gender segregation in the labour market. Moreover, Switzerland’s established youth panel study TREE as well as Bulgaria’s first-ever school leavers survey—which has been implemented in 2014 in collaboration with one of the authors—offer the necessary data for a comparative analysis. The consideration of Bulgaria, a country that seems to have achieved greater gender equality in education and employment opportunities than many other countries (Kovacheva 2008), also contributes to the hitherto limited research on school-to-work transitions and on the education–employment linkage in South Eastern Europe, as well as to insights into gender segregation in education and employment in a post-socialist country which has transitioned from a former planned socialist to a (liberal) market economy. Concretely, we want to test whether the different importance placed on the VET system in Switzerland and Bulgaria influences the strength of the linkage between education and employment, and (through this) the transmission of gender segregation into the labour market.

Below, we first present the educational systems and the labour market situations in Bulgaria and Switzerland. We then discuss institutional approaches to the explanation of gender segregation in education and employment. Thereafter, we present our data and the methodologies used in the analyses. Finally, we present and discuss our results, followed by some concluding remarks.

## The educational system and labour market situation in Switzerland and Bulgaria

### The Swiss and Bulgarian education systems compared

Comparable to its German and Austrian neighbours, the Swiss educational system has a strong vocational orientation. This is obvious from the high share of students pursuing vocational education at the upper secondary level, mostly acquired through company-based dual-track VET, which amounts to a good two-thirds of the age group (Wettstein et al. 2017, 100). Bulgaria’s education system, which combines some of the features from its socialist past with some new developments as it is the case with other Central European (CEE) countries (Rokicka et al. 2018), is also characterized by a relatively widely developed, largely school-based vocational education offer (Ilieva-Trichkova et al. 2015; Stefanova 2014). Still, the Bulgarian share of students enrolled in VET is (at 54%) below the level of Switzerland. The focus in Bulgaria is more strongly placed on general education. The share of students with a general upper secondary education (Grades 9–12) is 46%, compared to 34% in Switzerland (Imdorf et al. 2018).

More strongly vocationally structured educational systems are generally distinguished by greater stratification and higher vocational specificity (Shavit and Müller 2000). In Switzerland, stratification is already pronounced at the lower secondary level. Generally, children are assigned to different performance levels after primary school, with long-lasting consequences for their educational career. Buchmann et al. (2016) conclude that the access to the gymnasium as well as to higher education in Switzerland is significantly determined by the performance level to which a child is assigned at the end of primary school. They trace this back to the high stratification as well as to the low permeability of the Swiss educational system. The permeability between vocational and general education remains limited after the completion of upper secondary education. Whereas a general baccalaureate enables enrolment at university, students with a vocational education degree can acquire a Federal Vocational Baccalaureate (FVB), an extended and in-depth general education as part of a double qualification certificate, which allows them field-specific access to a university of applied sciences (UAS) (Wettstein et al. 2017). In Switzerland’s binary and mostly public higher education system, however, this vertical permeability between VET and tertiary education provides no direct access to more research-oriented university study.^1^ The tertiary education system also comprises professional colleges, which train professionals and managers and are accessible in the field of one’s initial vocational training without an FVB (ibid.).

In comparison with Switzerland, Bulgaria—where the division to different performance levels happens only at the transition to upper secondary education—has a less formal stratified education system. Lavrijsen and Nicaise (2012, 17) therefore refer to Bulgaria as a moderately stratified system (it has no lower secondary school tracking, and the age of first selection is 14) in contrast to Switzerland’s highly stratified system (with a tracked lower secondary school and an age of first selection of 12). In addition, greater lateral permeability between VET and higher education exists in Bulgaria (Imdorf et al. 2018; Boyadjieva and Ilieva-Trichkova 2018b). The majority of vocational education graduates receive an upper secondary education diploma, which allows them access to higher education (Ilieva-Trichkova et al. 2015), which is regulated by university entrance examinations (EP-Nuffic 2011). Bulgarian higher education consists of public and private universities, specialized universities, and higher education institutions, as well as colleges (Boyadjieva and Ilieva-Trichkova 2018a). Vocational colleges offer vocational education after secondary education in fields such as nursing, management, theatre, or tourism. They can be independent or part of universities, or there can be specialized higher education schools (Stefanova 2014). College education is more applied and less strongly focused on research compared to university education (EP-Nuffic 2011); as such, Bulgarian colleges may be considered functional equivalents of the universities of applied sciences in Switzerland.

If one compares the upper secondary education of both countries, vocational education in Switzerland produces noticeably more labour-market relevant qualifications than in Bulgaria. In Switzerland, dual-track VET, which is followed by 90% of all vocational students (Buchmann et al. 2016), combines company-based learning with lessons at vocational schools in 230 training programs. Due to the corporatist coordination of training companies, vocational schools, and occupational organizations, as well as its strong focus on practical education, Swiss VET produces very specific professional qualifications that are in tune with the needs of the labour market (Shavit and Müller 1998). As a consequence, the proportion of occupational changes one year after completing apprenticeship training is low, even though firm mobility is high. Müller and Schweri (2015) have shown that half of VET graduates continue to work at their training firms, 42% change firms but not their occupation, and 7% change both their firm and their learned occupation. In Bulgaria, in contrast to the high specificity of the Swiss VET system, a school-based vocational education system has increasingly been perpetuated and the relation with enterprises eliminated after the end of state socialism during which vocational education was part of the planned economy (Ilieva-Trichkova et al. 2015). A growing mismatch between labour demands and qualification offered by the education system has been identified a decade ago as a key reason for relatively high youth unemployment rates in Bulgaria (Council of Ministers 2007; Kostova 2008). Hence, even though the official list of professions for vocational education and training of the Bulgarian VET system lists some 500 specialities (qualifications) clustered in approx. 230 occupations (professions) (Cedefop 2018)—a number similar to the Swiss case—, training content is not always closely linked to modern job requirements, it does not sufficiently satisfy the needs of the labour market, and it is less well designed to convey specific professional qualifications (Stefanova 2014).

### Gender segregation in education and employment

Regarding gender in the educational system, the following picture emerges in comparing Bulgaria and Switzerland. Following educational expansion in both countries, women are now equally overrepresented in general upper secondary schools (female share: 56% in Bulgaria compared to 57% in Switzerland—Imdorf et al. 2018). Thereby, Bulgaria’s corresponding overrepresentation of young men in VET is representative for other CEE countries (Kogan et al. 2011). In terms of access to tertiary education, women have also caught up significantly. In Bulgaria, they make up 54% and in Switzerland 50% of all students who enrol in higher education. The educational advancement of women has, however, changed little regarding gender segregation in education. In Switzerland, high gender segregation has existed in vocational and tertiary education since the 1970s (Franzen et al. 2004; Leemann and Keck 2005). In Bulgaria, the situation looks different. While vocational education is relatively strongly gender segregated (Ilieva-Trichkova et al. 2015), higher education exhibits relatively low gender segregation both in terms of international comparison (Charles and Bradley 2009) and compared to other CEE countries (Kogan 2008). For example, Bulgarian women are exceptionally well represented in STEM (Science, Technology, Engineering, and Mathematics) compared to other European countries. In 2014, their share in Bulgaria accounted for 50%, while in Switzerland it stood at 32% (Imdorf et al. 2018).

Similarly, it can be assumed that there are different levels of horizontal gender segregation in the labour market in the two countries, although more recent comparative data on the question is sparse. Switzerland has risen to a middle position in occupational gender segregation among advanced industrial countries since the 1980s, when it still had one of the highest levels of horizontal gender segregation (Charles 2004). Bulgaria, on the other hand, having transitioned to a (liberal) market economy with a leading role of services after 1989 exhibits a comparatively low level of gender segregation in international comparison (Bieri et al. 2016). This can be traced back to the fact that women are more likely to be found in male-typed jobs in IT and technology (World Economic Forum 2013). The Bulgarian share of women in IT professions (28.5%) has been the highest in Europe (European Commission 2009). The lower gender segregation is partly a remnant from the time of state socialism where women’s labour force participation rate was among the highest in the world, and where occupational sex segregation did not reach the levels found in capitalist countries (Glass 2008). The socialist government guaranteed full employment to both genders in the 1980s and sought to reduce social and economic inequalities between the sexes in the labour market (Paskaleva et al. 1998), although in reality job segregation still did disadvantage women (Stoilova 2007).

## Institutional explanatory factors for gender segregation in education and employment

Gender segregation in education can be viewed as a potential cause for segregation in the labour market. Although this explanatory approach appears plausible or even obvious, previous research has paid little attention to it. Smyth and Steinmetz (2008) have investigated the connection between gender segregation in tertiary education and in the labour market and have shown that segregation in education and employment are strongly correlated within countries. The authors also point to the international variation of the connection, which suggests institutional factors in the design of the educational system or the labour market might influence the connection between segregation in education and employment.

To understand how patterns of horizontal gender segregation are transferred from education into the labour market—we call this process the *transmission* of gender segregation from education to employment—it is useful to highlight a few institutional circumstances of the educational system (Trappe 2006). One fact that might influence such a transmission is the strength of the linkage between the educational system and the labour market. Signal theory states that, the more clearly educational certificates communicate labour-market relevant information to the employer, the stronger the linkage strength between education and labour market (Steinmetz 2012). Different factors can influence the signal value of educational certificates, and thereby influence the linkage between education and employment. First, when educational paths within a country are standardized, employers assume that educational certificates reflect the abilities of applicants (Allmendinger 1989). Second, the stratification of the educational system affects the linkage between education and the labour market. Since in a stratified system educational certificates communicate more detailed and reliable information about the skills of an applicant, the linkage between education and work might increase with more stratification (ibid.). This linkage might therefore be more pronounced in Switzerland than in Bulgaria, where tracking occurs later and where the educational system is more permeable. Third, one must take the specificity of the skills learned in the vocational training into account. Maurice et al. (1992) offered one of the first studies investigating the relationship between education and work from an internationally comparative perspective. Comparing the German and the French labour markets, they distinguished two broad transition systems of vocational education and work organization: qualification spaces and organizational spaces (Maurice et al. 1992). In organizational spaces, vocational training conveys primarily general knowledge, and vocational skills are only acquired subsequent to education in the enterprise. The educational system is largely academically designed. In contrast, qualification spaces are distinct because they are structured by the vocational principle. Vocational training is strongly occupationally specified, with the goal of producing qualifications that can be used on the job (Shavit and Müller 1998). In qualification spaces, educational qualifications determine the first job placement, and most jobs are reserved for people with particular educational certificates (Charles and Buchmann 1994). Empirical evidence shows that, in systems with high vocational specificity (qualification spaces), people with the same educational certificate end up in a narrower group of jobs compared to educational systems that convey more general skills (organizational spaces). Thus, the linkage between education and work is stronger in systems with a pronounced vocational principle than in general educational systems (Shavit and Müller 1998).

Educational systems are distinguished by the extent to which they convey more general or specific occupational skills. Especially pronounced is the occupational specificity in countries with dual vocational education. Job specific skills are conveyed to the apprentices through vocational education acquired at the company, and they are canalized into set jobs after such training (Bol and Werfhorst 2013). The Swiss educational system is an example of such a strongly vocationally-oriented system that conveys specific occupational skills in a qualification space (Imdorf et al. 2017). In contrast to Switzerland, the Bulgarian educational system conveys more general skills, and vocational training is more school-based (Ilieva-Trichkova et al. 2015). Even though the Bulgarian VET system comprises hundreds of different learning occupations and specialities, there remains a mismatch between labour demands and vocational qualification. The fact that both the type of secondary education (e.g. arising from the different quality of upper secondary schools) and the institutional characteristics of higher education institutions strongly influence graduate employability (Boyadjieva and Ilieva-Trichkova 2015, 2018b) suggest that the Bulgarian education–employment transition system is more representative of organizational space. Therefore, it can be assumed that the linkage between education and work is stronger in Switzerland than in Bulgaria.

Even if vocational specificity and education–employment linkage are two important indicators of an education system organised through the vocational principle, these two dimensions do not necessarily need to be correlated in a strict sense. As already mentioned, the VET systems of Bulgarian and Switzerland consist of a comparable number of different training programs. Still, the education–employment linkage can be assumed less pronounced in the former compared to the latter. Likewise, the number of higher education programs is not necessarily a reliable indicator for the overall education–employment linkage of the higher education sector. Not only do many higher education programs offer rather broad and general skills. In addition, the strategic planning of university programs does not solely reflect employer needs and emerging industries, but it also builds on student demands. Hence, vocational specificity and education–employment linkage need to be distinguished with regard to their role in the transmission of gender segregation from education to employment.

The question then arises of the extent to which the different institutional designs of the educational system (vocational vs. general) influence the transmission of gender segregation into the labour market. It has been shown that gender segregation is already more pronounced in educational systems with a strong vocational principle than in general educational systems (Reisel et al. 2015). Empirical evidence for higher gender segregation in vocational educational systems is also presented in the study by Smyth and Steinmetz (2015). Their analysis shows that gender segregation is lower in countries where more general educational schooling is offered than in countries with a strongly developed vocational educational system. This connection has also been confirmed in the context of Switzerland. Cantons with more vocational educational opportunities have more gender-typical educational pathways and labour market entries than cantons that offer more general education at the upper secondary level (Imdorf et al. 2014).^2^ Finally, Solga and Konietzka (2000) and Trappe (2006) have pointed out that gender segregation can be transferred from education into the labour market when the linkage between education and work is regulated by the vocational principle.

In line with those research findings, we assume that the occupational specificity of educational offers which is especially prominent in the VET sector *enables* horizontal gender segregation in education (Kriesi and Imdorf forthcoming), whereas a strong education–employment linkage *supports the transmission* of gender segregation into the labour market. In contrast, the ‘non-transmission’ of such segregation may result from a weakened linkage which on the one hand allows employers to disregard the field of training or study of job candidates when they hire. On the other hand, graduates may look for employment in occupational sectors they have not specifically been trained for and thereby move to jobs which are less gender-typical than their previous educational trajectory. Based on this, we assume that, in countries where the vocational principle pre-structures schooling as well as the transition into the labour market (qualification space), the educational gender segregation more strongly transfers into the labour market than in countries where the vocational principle is less developed.

In summary, we hypothesize that the overall gender segregation in education is higher in Switzerland (which has a strongly vocationalized education system) than in Bulgaria (which has a more general and more academic educational system) (H1). Moreover, we hypothesize that the linkage between education and employment is stronger in Switzerland (which has a higher vocational specificity) than in Bulgaria (H2). Finally, we want to test the hypothesis that more distinct educational gender segregation in Switzerland more strongly transfers into the labour market than in Bulgaria because of the more pronounced linkage between education and work in the former country (H3).

## Data

As mentioned earlier, there are several reasons why we have chosen to compare the two cases of Bulgaria and Switzerland to test our hypothesis. Bulgaria and Switzerland both have a well-established and highly gender segregated vocational educational system, whereas the labour market can be expected to be more gender segregated in the latter case. The two education systems also differ in terms of their institutional configuration (vocational specificity, stratification, and assumed education–employment linkage). As we are interested in the effect of those institutional dimensions on gender segregation in the labour market, these two countries are well suited for a comparison. Moreover, they offer appropriate data to test our hypotheses.

Our case-study approach will not allow for statistical generalization of findings beyond the two cases under investigation. Like experiments, case studies are generalizable to theoretical propositions and not to an empirical universe (Yin 2014). The aim of our analytical strategy is therefore to find evidence for our theoretical arguments in order to advance theory building on gender segregation in education and employment. The findings from the case study may support or challenge our propositions.

To answer the question of how gender segregation is transmitted from education to employment in both countries, we use data from two comparable national youth surveys. For Switzerland, we use the nationally representative *TREE (Transition from Education to Employment)* panel study, which includes students who completed 9th grade in 2000 (at the age of 15/16) and who participated in the PISA study the same year. The individuals were surveyed a total of nine times between 2000 and 2014 and asked each time about their educational and work situation. We control for survey non-response as well as for the survey design (Sacchi 2013). For Bulgaria, we use the nationally representative *Bulgarian School Leaver Survey 2014 (BSLS 2014)*, which offers detailed information about educational careers and employment. The retrospective survey includes 15–35-year-olds who had completed their formal education at least one and at most 5 years prior to the survey date. Weights are available for survey design biases. Survey non-response was minimized by collecting the data in face-to-face encounters.

In both countries the sample has been restricted to individuals who (a) managed to transition from education to a first significant job (see details below) and (b) either graduated VET, university, or UAS (CH)/college (BG). Hence, individuals without upper secondary degree as well as those with a general baccalaureate (gymnasium)—which includes university dropouts—were excluded from the analyses (though these limitations do not apply to the dissimilarity analyses further down). The analysed Swiss sample includes 3215 cases (up to 3821 cases in the dissimilarity analyses) and the Bulgarian sample 885 cases (up to 1715 cases in the dissimilarity analyses). Listwise deletion was used to handle missing data in both countries.

Even though the Bulgarian data are based on a retrospective sample of cohorts born between 1980 and 2000 (whereas the Swiss data represents a prospective sample of cohorts born around 1985), the two data sets can be considered comparable for several reasons. First, the age distribution at the start of the first significant job is roughly the same in the two countries.^3^ Second, the retrospective Bulgarian survey is just as detailed in terms of the educational trajectories and the first significant job as the Swiss prospective survey. Whereas a major issue with retrospective data is memory error, we do not expect such errors regarding the remembered educational trajectory. Finally, we apply similar classification systems to identify educational programs (the International Standard Classification of Education 1997, or ISCED97) and jobs (the International Standard Classification of Occupations, or ISCO88).

To test the hypotheses, we require information about the level and specialty of educational programs, the first significant job, the distributions of men and women across educational programs and jobs (to measure gender segregation) and the representation of one sex within a program or job (to measure gender concentration). We take into account educational programs at the highest completed education level by applying the 2-digit ISCED97 classification.^4^ In addition to the educational content, we also take into account the educational sector of each program. In Switzerland, we distinguish between training received at an initial VET, at university, and at a UAS (including universities of teacher education). In Bulgaria, we distinguish between people with a vocational degree, with a university degree, or with a college degree. Our measure for education is composed of the 2-digit ISCED information on the educational content, as well as by an additional digit on the educational sector.

As occupation of interest, we use the first significant job for each graduate. The latter is defined as the first job of at least 6 months duration secured after the end of education. For the operationalization of the first significant job, we use the two-digit codes of the ISCO88 classification.

In order to measure gender segregation, we analyse the distribution of men and women across educational programs and jobs according to the two youth surveys (based on respective dissimilarity indices, see below). Gender concentration of education and the first significant job refers to the representation of women and men respectively within an educational program or occupational category (Siltanen et al. 1995). It is defined as the share of people of their own gender in their respective educational/occupational category and is computed based on official national educational and labour market statistics for Bulgaria and Switzerland (see Appendices 1 (Tables 10, 11, 12) and 2 (Table 13) for the respective shares of females).

## Methods

To test the first hypothesis (*gender segregation in education is higher in Switzerland compared to Bulgaria*), we calculated dissimilarity indices following Duncan and Duncan (1955).^5^ What is calculated is the proportion of men plus the proportion of women who would need to change their occupation (or their educational program) to have the same proportion of women in every occupation (program) (Anker 1998, 90). The index takes the value of 0 when men and women are evenly distributed across occupations/educational programs. The index reaches the maximum value of 1 when all occupational groups/educational programs are filled with only men or women (Busch 2013).

To analyse the second hypothesis (*the linkage between education and employment is stronger in Switzerland compared to Bulgaria),* a measure for the association between school-leaving credentials and subsequent labour market position is required (this is referred to as *linkage strength* in the following). Based on a recent analysis of school-to-work linkages in the United States, Germany, and France by DiPrete et al. (2017), a strong linkage is assumed when individuals with the same educational credentials cluster in a relatively small number of occupations. On the other hand, if individuals with a given educational credential are located in many different occupations, we observe a weak linkage. This theoretical consideration can be measured using entropy-based segregation measurements (Mora and Ruiz-Castillo 2011). Entropy measures are based on the amount of additional information gained by knowing a particular attribute of an individual. For example, entropy-based measures of the education–occupation linkage reflect gains in the ability to predict an occupation if one knows the individual’s educational category. That is, if individuals with a given educational credential cluster in a relatively small number of occupations, the ability to predict one’s occupation by knowing his or her educational category increases significantly. In contrast, if individuals with a given educational credential transition into a wide range of different occupational categories, the ability to predict an individual’s occupation by knowing his or her educational category hardly improves.

An example for an entropy-based segregation measurement is the *Mutual Information Index* (M), which—due to its decomposability—will be used in the present analysis to measure linkage strength:

$$\begin{aligned} M = \sum\limits_{j = 1}^{J} {p_{j} \left( {T(P_{j} ) - T(P_{j\left| g \right.} )} \right)} \hfill \\ \hfill \\ \end{aligned}$$

If *P*_*j*_ reflects the distribution of individuals over the occupational categories *j* and *T(P*_*j*_*)* the entropy of the occupations, *M* is defined as the average reduction in entropy between its total value *T(P*_*j*_*)* and its value within a given educational category *T(P*_*j|g*_*),* averaged over the occupational categories.^6^ *M* can be interpreted as follows: The higher the Mutual Information Index (i.e. the higher the linkage strength), the higher the share of individuals of a given educational category who cluster in the most common occupations of this educational credential.^7^

As already mentioned, the main advantage of the Mutual Information Index is its decomposability. First, *M* can be decomposed into local linkages. The contribution of a given educational category (or a group of educational categories, in other words an educational sector) to the total linkage strength of a country can thereby be assessed. The present analysis makes use of this decomposition to analyse the contribution of different educational sectors (Vocational Education, University, UAS/College) to the overall linkage strength of a country.

A second form of decomposition addresses an issue that affects every entropy-based measurement. *M* is influenced by the distribution of individuals over the educational and occupational categories. Hence, differences in *M* between countries are partly the result of differences in the marginal distribution of individuals over the educational and occupational categories. The present contribution, however, focuses on the *structural* linkage strength between education and occupation (i.e. adjusted for differences in the educational and occupational distribution) rather than on *overall* linkage strength, which might be influenced by the distribution of individuals across educational and occupational categories. To counter this problem, country differences in *M* are decomposed into three parts, of which one is composition invariant and will be used in the present analysis (see Appendix 3).

For the third hypothesis (according to which gender segregation in education is more strongly transmitted into employment in Switzerland than in Bulgaria due to the more pronounced linkage between education and work in Switzerland), we calculate, separately for men and women and for both countries, multivariate Ordinary Least Squares (OLS) regressions with gender concentration of the first significant job as the dependent variable.^8^ The interaction term between gender concentration of the educational program and the linkage strength (M) described above, together with the main effect of the respective gender concentration variable are our measures for the transmission of gender segregation from education to the labour market. The linkage between education and employment is subdivided in local link components per educational program at the ISCED97 one-digit level and the occupations at the ISCO88 one-digit level. We thus have an individual measure of the linkage strength between education and employment for each person.^9^

### Control variables

Since various studies point out that the educational level of parents is negatively related to gender stereotypical role models (Davis and Greenstein 2009), we introduce the educational level of parents into the model, as well as the student’s language competency (reading performance in Switzerland; language grades in Bulgaria) to proxy for individual educational attainment.^10^ A further control variable for Switzerland is language region, and for both countries a distinction is made between urban and rural areas, since it is possible that the structure of the labour market (as well as the labour market chances) vary regionally.^11^

All the analyses are weighted with an individual sampling weight that adjusts for survey design biases in both countries and for panel mortality in Switzerland. Table 1 displays the operationalization, scales, and distribution of the used variables.

**Table 1:** Descriptive statistics of used variables. Source: Own calculations

	Variable	Measurement	Scale	Distribution
DV	Gender concentration: first significant job	Proportion of own gender in first significant job (ISCO88 2-digit)	0–100%	Mean: CH: 62.56 BG: 57.42
IV	Gender concentration: educational program	Proportion of own gender in educational program (ISCED97 2-digit)	0–100%	Mean: CH: 71.44 BG: 57.34
	Linkage strength	Mutual Information Index (ISCED97 1-digit and ISCO88 1-digit)	CH: 0–3.5 BG: 0–3.2	Mean: CH: 0.15 BG: 0.12
	Educational sector	Distinction between VET, University, and UAS (CH) resp. College (BG)	1 = VET 2 = University 3 = UAS/College	–
CV	Parental education	Tertiary education parents: yes/no	1 = yes 0 = no	CH: 1: 34% 0: 66% BG: 1: 24.6% 0: 75.4%
	Language skills	Literacy PISA 2000 (CH) Language grades at school (BG)	1 = low 0 = medium/high	CH: 1: 24.2% 0: 75.8% BG: 1: 63.3% 0: 36.7%
	Region	Urban/rural	1 = rural 0 = urban	CH: 1: 36.3% 0: 63.7% BG: 1: 46.7% 0: 53.3%
	Language region	Latin/German (CH)	1 = German 0 = Latin	CH: 1: 51.8% 0: 48.2%

DV: dependent variable; IV: independent variables; CV: control variables

## Results

### Gender segregation in education

Table 2 shows gender segregation in education and in employment measured by the dissimilarity index. It is apparent that education in Switzerland is more strongly gender segregated than in Bulgaria. To achieve an equal distribution in education, 56% of men and women would have to change their educational program in Switzerland, while in Bulgaria only 31% would have to do so. When we compare gender segregation separately for the three educational sectors, the differences between Bulgaria and Switzerland become smaller. This is partly because a bigger share of students (34.2%) complete a general upper secondary school in Bulgaria without attending or completing tertiary education compared to Switzerland (6.3%; this educational group is not listed separately in Table 2, but it is included in the column ‘Education: Total’). This group shows only low levels of gender concentration (female share in general upper secondary school: 50% in BG and 55.7% in CH) with which the overall gender segregation in education is reduced (this applies more strongly to BG than to CH). Table 2 also shows that vocational education as well as the university sector are more strongly gender segregated (by about 5% each) in Switzerland compared to Bulgaria. The highest gender segregation can be found in UAS in Switzerland and in the colleges in Bulgaria. In Bulgaria, however, the high segregation of the colleges does not have a very big impact on overall gender segregation in education, since only a few people (3.9%) complete a college course. The differences in gender segregation in employment (the first significant job) are a bit bigger between the two countries. Further descriptive analyses have shown that the relatively high gender segregation in the Swiss labour market can mostly be attributed to the gender-typed occupations of men. In contrast, the lower gender segregation in Bulgaria is explained primarily by the fewer gender-typed jobs for men in Bulgaria.

**Table 2:** Gender segregation in education and employment (Index of dissimilarity). Source: Own calculations

	Education: total	VET	University	UAS (CH), Colleges (BG)	Labour market (1st job)
Switzerland	0.557 (O: 3418; C: 123)	0.592 (O: 1746; C: 33)	0.420 (O: 670; C: 42)	0.576 (O: 561; C: 47)	0.576 (O: 3821; C: 98)
Bulgaria	0.314 (O: 1715; C: 128)	0.546 (O: 557; C: 46)	0.372 (O: 446; C: 51)	0.674 (O: 66; C: 28)	0.452 (O: 1306; C: 101)

Education: ISCED97 3-digit; Labour market: ISCO88 3-digit. O: Number of observations; C: Number of categories

In summary, the first hypothesis (H1) can be confirmed: education is indeed less strongly gender segregated overall in Bulgaria than in Switzerland. However, when we consider the different educational sectors separately, the differences are relatively small.

### Linkage of education and first significant job

**Fig. 1:**
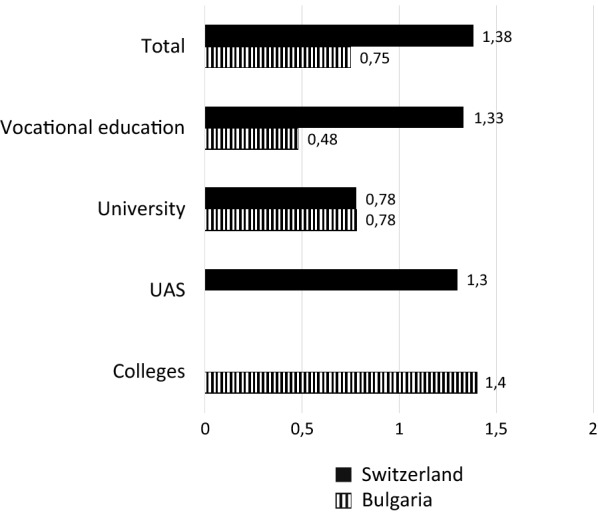
Linkage strength between education and early employment, by educational sector (Mutual Information Index) (Source: Own calculations)

Our second hypothesis is that the linkage between education and early employment is stronger in Switzerland than in Bulgaria. To test this, we first determine the Mutual Information Index *M* (operationalization of the linkage strength) separately for the different educational sectors. In a second step, we calculate the composition-invariable term of the index to compare the structurally conditioned linkage for Switzerland and Bulgaria. Figure 1 shows the Index *M* for both countries. It is apparent that the overall linkage strength is distinctly higher for Switzerland than for Bulgaria.

When the total linkage strength is subdivided into the local linkage strengths for the respective three educational sectors, it becomes evident that the differences in the total linkage strength between the two countries emerge in particular due to the differences in the linkage strength of vocational education and employment. In Switzerland, young workers with vocational education display a very high linkage strength, while in Bulgaria it is relatively low. In contrast, the linkage strength for workers with university education are identical in both countries. The value of *M* for people with vocational education in Switzerland is 1.33. That means that, on average, 46.6% (0.2 * 1.33 + 0.2) of all graduates with a VET degree in Switzerland transition into the three most common occupational fields. In Bulgaria, this share stands at only 29.6% (0.48 * 0.2 + 0.2).

**Fig. 2:**
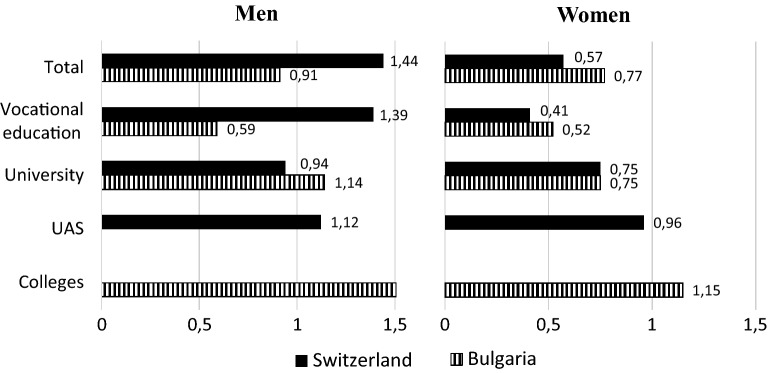
Linkage strengths by educational sector and gender (Mutual Information Index) (Source: Own calculations)

When we consider the linkage of education and employment separately by gender (Fig. 2),^12^ it becomes evident that the stronger link in Switzerland emerges primarily due to men. Men in Switzerland exhibit a particularly tight linkage between education and work. Especially strong is the linkage *M* for men with vocational education (1.39) or with a degree from a UAS (1.12). In contrast, the linkage for men with university education in Switzerland is weaker (0.94). The opposite is the case for men in Bulgaria. Here, the linkage for people with a university degree (1.14) is stronger than for people with a vocational education (0.59). When looking at the results for women, it is apparent that their linkage strengths are generally lower than for men, especially in Switzerland. The linkage is particularly weak for women with vocational education in Switzerland. In contrast, the linkage strength for women with a UAS degree is especially high. In Bulgaria, the strongest linkage is for men and women with a college degree (this is also the case for female UAS graduates in Switzerland).

The results thus far appear to partially support the second hypothesis: for men at least, the linkage strengths appear to be higher in Switzerland than in Bulgaria. As previously mentioned, the distribution of the educational and occupational categories influences the Mutual Information Index. The question now arises of whether the higher linkage strengths in Switzerland can indeed be ascribed to a tighter connection between education and work, or whether the different distribution of people in the educational and occupational categories might in fact be responsible. To address this question, we carry out a decomposition of the difference in the linkage strength between the two countries. By doing so, we can calculate the composition-invariable part of the country difference of the linkage strength, which is necessary to clarify the second hypothesis.

Table 3 shows the decomposition of the country difference for the entire educational system, as well as for the educational sectors VET and University.^13^ The first column in Table 3 (*M*_CH_ − *M*_BG_) shows the raw difference in the linkage strength between Switzerland and Bulgaria, based on the results in Fig. 1. A positive value signifies a higher linkage strength for Switzerland and a negative value a higher linkage strength for Bulgaria (in Table 3 all respective values are positive). ΔN is the composition invariable term. ΔE and ΔO reflect the part of the country difference in the linkage strength that emerges due to the different distribution of the educational categories (ΔE) and of the occupational categories (ΔO). ΔN shows that the composition invariable country differences are even bigger than the raw differences for all three categories (Total, VET, University). This means that, without decomposition, the respective country differences are underestimated. Based on this, we conclude that Switzerland and Bulgaria are indeed structurally different through the dissimilar strengths of their linkage between education and work (there is a stronger connection in Switzerland). ΔE and ΔO can be interpreted as follows: Positive values indicate an overestimation in the sense of an artefact in the global difference of the raw linkage strength between the two countries through compositional differences in the educational programs (ΔE) or in the occupational categories (ΔO). Negative values indicate that the raw country differences in the linkage strength are underestimated.^14^ The raw linkage strength *M*_CH_ − *M*_BG_ is always the sum of ΔN, ΔE and ΔO. From Table 3, we can also conclude that the total country difference in the linkage strength is largely due to the differently strong linkage of people with a vocational education. In contrast, the difference in the linkage strength for people with a university degree is less pronounced (but here, too, the structural linkage to jobs in Switzerland is stronger than in Bulgaria).

**Table 3:** Decomposition of the country differences in the Mutual Information Index: total. Source: Own calculations

	*M*_CH_ * − M*_BG_	ΔN	ΔE	ΔO
Total	0.62	0.67	0.29	*− *0.34
VET	0.86	0.97	*− *0.01	*− *0.1
University	0.0008	0.2	*− *0.09	*− *0.29

*M*_CH_ − *M*_BG_: (*M* for Switzerland) − (*M* for Bulgaria), ΔN: (composition-invariant part Switzerland) − (composition-invariant part Bulgaria), ΔE: (educational entropy Switzerland) − (educational entropy Bulgaria), ΔO: (occupational entropy Switzerland) − (occupational entropy Bulgaria)

Figure 2 has shown that the linkage strengths differ by gender. For this reason, we conduct an additional decomposition separately by gender. Table 4 shows the decomposition of the linkage strengths for men, and Table 5 for women. What is notable is that the raw country difference between the linkage strengths in Bulgaria and Switzerland (*M*_CH_ − *M*_BG_) is negative for men with a university degree. A positive value of 0.19 is, however, obtained once we adjust the country difference by the distribution of the educational and job categories. This means that the linkage strength is also stronger in Switzerland than in Bulgaria for men with university degrees. What is nevertheless confirmed is that the country difference in the linkage strength for men with vocational education is markedly higher.

**Table 4:** Decomposition of the country differences in the Mutual Information Index: men. Source: Own calculations

	M_CH_* − *M_BG_	ΔN	ΔE	ΔO
Total	0.52	0.78	0.39	*− *0.64
VET	0.81	1.04	0.17	*− *0.4
University	*− *0.19	0.19	0.09	*− *0.49

*M*_CH_ − *M*_BG_: (*M* for Switzerland) − (*M* for Bulgaria), ΔN: (composition-invariant part Switzerland) − (composition-invariant part Bulgaria), ΔE: (educational entropy Switzerland) − (educational entropy Bulgaria), ΔO: (occupational entropy Switzerland) − (occupational entropy Bulgaria)

**Table 5:** Decomposition of the country-differences in the Mutual Information Index: women. Source: Own calculations

	*M*_CH_ * − M*_BG_	ΔN	ΔE	ΔO
Total	*− *0.19	0.22	0.04	*− *0.46
VET	* − *0.11	0.31	*− *0.28	*− *0.14
University	0.003	0.24	0.01	* − *0.25

*M*_CH_ − *M*_BG_: (*M* for Switzerland) − (*M* for Bulgaria), ΔN: (composition-invariant part Switzerland) − (composition-invariant part Bulgaria), ΔE: (educational entropy Switzerland) − (educational entropy Bulgaria), ΔO: (occupational entropy Switzerland) − (occupational entropy Bulgaria)

The country differences in the structural linkage strengths also changes notably for women (Table 5) after the decomposition. As soon as we control for the fact that women in Switzerland are more often in jobs with a weak linkage to the educational system (compared to Bulgaria),^15^ the composition invariant link (ΔN) proves also to be stronger for women with vocational education in Switzerland (compared to Bulgaria). In comparison to men, however, the country differences are smaller between women, especially for those with vocational education.

In summary, we observe that the biggest difference between the countries concerns men with vocational education. For this group, Bulgaria exhibits a rather weak linkage of education and work, while the linkage is very strongly pronounced for men with vocational education in Switzerland. For men and women with a university degree, the country difference turns out to be comparatively small. We thus find support for hypothesis 2: especially after adjusting for country differences by the distribution of educational and job categories, we find markedly stronger linkage between education and work for Switzerland than for Bulgaria.

In a final step, in order to better understand the role of VET in the transmission of gender segregation from school to work, we want to answer the question of whether gender segregation in education is more strongly transferred into employment in Switzerland than in Bulgaria due to the more pronounced linkage of education and employment in Switzerland (hypothesis 3).

### The influence of gender concentration in education and linkage strength on occupational gender concentration and the transmission of gender segregation from school to work

Table 6 presents the results of the multivariate linear regression model for men in Switzerland. The dependent variable measures gender concentration (the share of men) in the first job. As independent variables, we analyse the educational sector, the share of men in education (divided by 10),^16^ the linkage strength of the educational sector, its interaction with the gender concentration in education, as well as several control variables (family educational background, literacy, geography, and language region).

**Table 6:** Influence of educational gender concentration and linkage strength on occupational gender concentration for men in Switzerland (OLS, average marginal effects). Source: Own calculations

Occupation: % of men	Model 1	Model 2	Model 3
Educational sector (ref. VET)			
UAS/PH	− 4.682** (1.733)	− 1.421 (1.749)	0.642 (1.784)
University	− 21.09*** (2.756)	− 10.70*** (2.129)	− 7.991*** (2.169)
% men in educational program		3.131*** (0.283)	3.288*** (0.283)
Linkage strength		− 13.85 (10.15)	− 15.23 (10.16)
% men in educ. progr. * linkage strength		5.104*** (1.183)	5.052*** (1.184)
Parental education: tertiary			− 2.395* (0.933)
Literacy: low (ref. high)			3.743** (1.147)
Region: rural (ref. urban)			1.495 (1.033)
Language region: German (ref. Latin)			2.426 (1.238)
*N*	1362	984	984
Adj. *R*^2^	0.043	0.484	0.496

Gender concentration in education: % of men in educational program/10 (ISCED97 2-digit); occupational gender concentration: % of men in first significant job (ISCO88 2-digit); linkage strength: Mutual Information Index. Standard errors in parentheses; *p < 0.05, **p < 0.01, ***p < 0.001

Model 1 shows that men with a tertiary degree (university or UAS) transition into first jobs with a significantly lower gender concentration than men with a vocational degree. Compared to men with vocational education, men with a university degree (UAS degree) transition into jobs with an (on average) 21 (5) percentage points smaller share of men. In Model 2, we also include the gender concentration of the educational program, the linkage strength, and the respective interaction. What becomes apparent is that the educational sector UAS loses its significance, which means that the negative influence of UAS on gender concentration in the labour market is the result of the lower gender concentration in the education of men with a UAS degree compared to those with a vocational education. Furthermore, it is shown that gender concentration in the educational program has a significant positive influence on the gender concentration of a job. If the share of men in education increases from 0 to 10 percentage points, the share of males in the first job increases by three percentage points. We also see that the interaction between the linkage strength and gender concentration of education has a statistically significant positive influence on the gender concentration of a job. The influence of gender concentration in education on the job increases with increasing linkage strength. Model 3 also controls for the educational background, literacy, geography, and regional differences. Significant effects appear for educational background and reading achievement: in Switzerland, men who have parents with a higher education degree controlling for their educational career transition into occupations with a slightly lower gender concentration (difference: 2.4 percentage points) compared to the reference group. Men with a low reading achievement transition into occupations that on average have a somewhat higher male share (3.7 percentage points). The findings from the previous models, however, do not change.^17^ The results support our third hypothesis for men in Switzerland.

Table 7 displays the results for the multivariate regression for women in Switzerland. As for the men, it is shown that women with a university degree transition into jobs with a lower gender concentration than those with a vocational degree. In contrast to the men, women with a UAS degree transition into occupations with a higher gender concentration (Model 1). Further to this, and also regarding the influences of gender concentration of education and linkage strength (Model 2), we observe similar results for women as for men, although the interaction effect is stronger for women. When we consider the control variables (Model 3), we see that women whose parents have a higher education degree also transition into occupations with on average a 2.3 percentage points lower female share in comparison to women without such parents. The third hypothesis is thus also supported for women in Switzerland.^18^

**Table 7:** Influence of educational gender concentration and linkage strength on occupational gender concentration for women in Switzerland (OLS, average marginal effects). Source: Own calculations

Occupation: % of women	Model 1	Model 2	Model 3
Educational sector (ref. VET)			
UAS/PH	3.353* (1.383)	− 0.725 (1.288)	− 0.174 (1.375)
University	− 4.810** (1.741)	− 5.128*** (1.250)	− 5.013*** (1.333)
% women in educational program		2.684*** (0.289)	2.379*** (0.298)
Linkage strength		− 66.63*** (5.814)	− 68.67*** (5.918)
% women in educ. progr. * linkage strength		13.08*** (0.844)	13.32*** (0.866)
Parental education: tertiary			− 2.277** (0.853)
Literacy: low (ref. high)			− 2.674 (1.466)
Region: rural (ref. urban)			0.825 (0.990)
Language region: German (ref. Latin)			− 0.418 (1.129)
*N*	1853	1037	1032
Adj. *R*^2^	0.007	0.521	0.523

Gender concentration in education: % women in educational program/10 (ISCED97 2-digit); occupational gender concentration: % women in first significant job (ISCO88 2-digit); linkage strength: Mutual Information Index. Standard errors in parentheses; *p < 0.05, **p < 0.01, ***p < 0.001

Next, we test whether educational gender concentration also has a stronger impact on gender concentration for those occupations that are more strongly linked to particular educational fields in Bulgaria. Table 8 shows the multivariate regression results for men. We again observe that men with a university degree transition into occupations with a lower gender concentration than those with a vocational degree. For men with a college degree, no significant effects are obtained. Once we control for the educational gender concentration in Model 2, the effect of the university degree no longer shows significance. On the other hand, the educational programs with a higher share of males have a significant positive effect on the male share in the first job. The lower occupational gender concentration of people with university degrees can thus primarily be explained by the fact that these people complete programs with a lower gender concentration compared to their male peers who pursue a vocational education. In contrast to Switzerland, the influence of the educational gender concentration on the first job does not increase with a stronger linkage (there is a non-significant interaction between linkage strength and male share in education). We thus do not find support for the third hypothesis for men in Bulgaria.

**Table 8:** Influence of educational gender concentration and linkage strength on occupational gender concentration for men in Bulgaria (OLS, average marginal effects). Source: Own calculations

Occupation: % of men	Model 1	Model 2	Model 3
Educational sector (ref. VET)			
University	− 10.50*** (2.556)	0.225 (2.819)	− 0.878 (3.372)
College	3.620 (5.062)	4.442 (5.435)	3.159 (5.666)
% men in educational program		3.027*** (0.653)	3.041*** (0.662)
Linkage strength		1.463 (23.04)	− 1.181 (23.09)
% men in educ. progr. * linkage strength		2.594 (3.720)	3.041 (3.747)
Parental education: tertiary			4.792 (3.345)
Literacy: low (ref. high)			− 1.866 (3.279)
Region: rural (ref. urban)			1.165 (2.797)
*N*	436	311	309
Adj. *R*^2^	0.044	0.143	0.146

Gender concentration in education: % men in educational program/10 (ISCED97 2-digit); occupational gender concentration: % of men in first significant job (ISCO88 2-digit); Linkage strength: Mutual Information Index. Standard errors in parentheses; *p < 0.05, **p < 0.01, ***p < 0.001

Finally, results for women in Bulgaria are presented in Table 9. In Model 1, we do not find effects of the educational sector on gender concentration of the first job. Model 2 shows that the effect of educational gender concentration on gender concentration in the labour market increases with growing linkage strength for women in Bulgaria in contrast to men. The main effect of educational gender concentration loses its significance once we control for the interaction term with the linkage strength. The significant interaction effect remains even after controlling for educational variables and urban–rural differences in Model 3.

**Table 9:** Influence of educational gender concentration and linkage strength on occupational gender concentration for women in Bulgaria (OLS, average marginal effects). Source: Own calculations

Occupation: % of women	Model 1	Model 2	Model 3
Educational sector (ref. VET)			
University	3.588 (1.851)	3.498 (2.057)	2.856 (2.385)
College	− 0.257 (3.733)	− 1.042 (3.879)	− 1.373 (4.108)
% women in educational program		0.753 (0.636)	0.742 (0.660)
Linkage strength		− 73.48*** (22.01)	− 75.66** (24.00)
% women in educ. progr. * linkage strength		9.421** (3.298)	9.715** (3.621)
Parental education: tertiary			2.749 (2.314)
Literacy: low (ref. high)			− 0.417 (3.688)
Region: rural (ref. urban)			− 0.959 (2.076)
*N*	449	363	358
Adj. *R*^2^	0.006	0.069	0.066

Gender concentration in: % women in educational program/10 (ISCED97 2-digit); occupational gender concentration: % of women in first significant job (ISCO88 2-digit); linkage strength: Mutual Information Index. Standard errors in parentheses; *p < 0.05, **p < 0.01, ***p < 0.001

In summary, the third hypothesis is supported with the exception of Bulgarian men: the effect of educational gender concentration on gender concentration of the first job increases with a stronger linkage between education and employment. Linkage strength contributes to the transmission of gender segregation from education to employment. However, the predictive power of the models differ strongly between Switzerland and Bulgaria. While the models for men and women in Switzerland explain 50% respectively 52% of the variation in the dependent variable, this percentage is much lower in Bulgaria, with 15% (men) and 7% (women) respectively. The educational gender concentration and the linkage strength appear to be much stronger explanatory factors for the gender concentration in the labour market in Switzerland than in Bulgaria.^19^

## Discussion

The goal of the present study was to analyse the role of vocational education in the transmission of educational gender segregation into the labour market. We compared Switzerland and Bulgaria because gender segregation and the institutional features of the educational systems differ between the two countries. Bulgaria exhibits lower gender segregation in higher education and in the labour market than Switzerland does. The Bulgarian educational system is less occupationally-oriented, which can be observed in the lower share of people with vocational education as well as in the reduced vocational specificity of its education. Specifically, we analysed whether the differences in the degree of occupational orientation of the educational system affect the linkage between education and employment, and thereby also the transmission of educational gender segregation into the labour market.

Based on the distinction of qualification space (education systems with high vocational specificity), organizational space (educational systems that convey more general skills), and assumptions on how institutional dimensions impact on gender segregation in education and employment, we formulated and tested three hypotheses using data from national youth surveys. First, we assumed that overall horizontal gender segregation in education is higher in Switzerland than in Bulgaria due to the greater importance of vocational education in the former country. Dissimilarity indices have confirmed those assumptions about higher gender segregation rates in Switzerland.

Second, we assumed that the linkage strength between education and employment would be stronger in Switzerland than in Bulgaria because of the stronger labour market orientation in Switzerland, which is realized through occupationally structured connections between educational programs and jobs. Based on a recent study by DiPrete et al. (2017), we calculated a Mutual Information Index to measure the linkage strength between education and the labour market. The second hypothesis was also confirmed. The results showed that the linkage strength is particularly strong for individuals with vocational education in Switzerland, while the linkage strength is much weaker for people with university degrees. In Bulgaria, however, we observed the reverse: people with a university degree exhibited stronger linkages than those with vocational education. This suggests that tertiary degrees offer higher signal values to employers in Bulgaria. We also observed that the differences between Switzerland and Bulgaria were bigger for men than for women. Thus, the biggest country differences were confirmed for men with vocational education (there was a very strong linkage between education and employment in Switzerland, as opposed to a weak linkage in Bulgaria).

Third, we tested the theoretical assumption that gender segregation in education more strongly transfers into the labour market where there is a more pronounced linkage between education and employment. With the exception of Bulgarian men, our results support a significant positive interaction effect of educational gender concentration and the linkage strength on gender concentration in the labour market. The stronger the relation between education and employment, the higher the transmission of gender segregation from school to work.

To sum up, the following picture emerges for Switzerland, a country that represents a qualification space: for men with vocational degrees, educational gender segregation and the linkage between education and work is higher than for men with a university degree. The stronger linkage between vocational education and the labour market transfers the distinct gender segregation in VET even more strongly into the labour market than the less pronounced gender segregation in university education. As such, we were able to confirm an institutional explanation for the extensive occupational gender segregation for men in the Swiss labour market. For women, it is primarily the relatively high gender segregation in the university of applied science sector and the segregation in the university sector that transfer into the labour market due to the linkage dynamics.

For men in Bulgaria—a country rather conceived as an organizational space—the linkage of education and employment (notably weaker than in Switzerland) does not affect the transmission of gender segregation into the labour market. This means that the higher gender segregation in vocational education does not transfer more strongly into the labour market than the relatively lower segregation levels at Bulgarian universities. This may help explain the lower levels of gender segregation (compared to Switzerland) in the Bulgarian labour market at the job-entry stage. For women in Bulgaria, the linkage between education and work significantly affects the transmission of educational gender segregation into the labour market. For them, lower gender segregation at the universities more strongly translates into the labour market than the higher segregation in vocational education, which in turn may also help explain the lower overall gender segregation in the Bulgarian labour market compared to the Swiss case.

However, the explanatory power of the Bulgarian models remains low compared to the Swiss models. In future analyses, it will therefore be important to identify other relevant factors to explain gender segregation in the Bulgarian labour market. Moreover, our theoretical framework, which points to the different degree of stratification and vocational specificity in the two educational systems of Switzerland and Bulgaria, does not provide any explanation for the gender differences with regard to the linkage strength within and across countries. Further research will be needed to better understand why the linkage between education and employment is generally more pronounced for men, especially in Switzerland.

The present study faces some additional limitations that should be considered for further research. Due to limited sample sizes, our analysis is based on ISCED and ISCO 2-digit classifications, which are accompanied with reduced information on gender segregation in education and employment due to aggregation. Furthermore, as mentioned earlier, we did not control for takeover effects, i.e. whether the training companies hire their apprentices at the end of their vocational training in the Swiss case. Even though a majority of VET graduates who change company at the end of training remain in their learned occupation (Müller and Schweri 2015), we do not know if firm mobility results in a similar transmission of gender segregation from education to early employment as it does in the case of firm takeover.

With respect to the generalizability of our findings, a two-case country study only allows for analytic generalization (Yin 2014). Additional future case studies on countries that represent either a qualification space or an organizational space are required to replicate our findings and to better understand the gendered nature of different school-to-work transition systems. As there is strong variation between national education systems and their interrelation with labour market structures, further research is needed to determine whether our results hold for other countries.

This study sought to formulate and empirically test education–institutional explanations for the relatively high horizontal gender segregation in the Swiss labour market through a country comparison with Bulgaria. We conclude that a vocationally dominant education system like that of Switzerland does not merely ‘produce’ a high degree of gender segregation in education; it also transmits the segregation more strongly into the labour market than in a system that is more oriented toward general education and less closely tied to the demands of the labour market, such as the one that exists in Bulgaria. This is a new finding in the research field of education and gender segregation in employment.

Given that a strong linkage between education and work is efficient from an economic perspective and is also desirable for the graduates (since it guarantees a predictable and educationally adequate transition into the labour market), the question of the practical relevance of our study arises. It is not reasonable to suggest that educational and labour market policies should aim to reduce the linkage between education and work to allow for more gender equity in the labour market. Rather, the high importance of vocational education in the creation (Reisel et al. 2015) and transmission of gender segregation in the employment system points to the need for policies that focus on the reduction of gender segregation in (vocational) education. One strategy might be to allow for more vocational permeability in the educational system and to facilitate the vocational reorientation of young men and women through affordable second-degree options or, alternatively, by promoting the permeability between vocational and university education. The latter is at least an option for men to conclude their educational careers in a less gender-typical manner, as has been shown in a recent study that also compares Switzerland and Bulgaria (Imdorf et al. 2018).

## Appendices

### Appendix 1

See Tables 10, 11, 12.

**Table 10:** Classification of educational programs (ISCED97 2-digits) and share of women per educational program in Bulgaria and Switzerland: Initial Vocational Education and Training (VET)

ISCED	Description	% of women in educational program^a^	
		Bulgaria	Switzerland
21	Arts	62.7%	59.9%
32	Journalism and information	n.a.	72.3%
34	Business and administration	66.4%	68.6%
48	Computing	49.5%	8.9%
52	Engineering and engineering trades	15.1%	4.6%
54	Manufacturing and processing	58.2%	27.0%
58	Architecture and building	27.8%	11.2%
62	Agriculture, forestry, and fishery	22.9%	22.4%
64	Veterinary	47.9%	98.4%
76	Social services	74.8%	89.5%
81	Personal services	56.6%	78.6%
84	Transport services	18.9%	18.3%
85	Environmental protection	68.1%	2.9%
86	Security services	25.2%	32.2%

^a^Data sources: Bulgarian National Statistical Institute, pupils enrolled in professional classes in state, municipal, and private upper secondary schools, nationally pooled data for 2005/6–2013/14 (Bulgaria); Swiss Federal Statistical Office, pooled statistics of upper secondary level diplomas 2000–2010 (Switzerland)

**Table 11:** Classification of educational programs (ISCED97 2-digits) and share of women per educational program in Bulgaria and Switzerland: Universities

ISCED	Description	% of women in educational program^a^	
		Bulgaria	Switzerland
14	Teacher training and education science	71.6%	75.4%
21	Arts	62.2%	74.1%
22	Humanities	69.2%	59.1%
31	Social and behavioural science	62.6%	56.1%
32	Journalism and information	71.1%	n.a.
34	Business and administration	63.5%	32.4%
38	Law	57.6%	53.0%
42	Life sciences	72.7%	52.4%
44	Physical sciences	56.4%	32.9%
46	Mathematics and statistics	47.6%	29.8%
48	Computing	35.4%	12.7%
52	Engineering and engineering trades	25.9%	9.7%
54	Manufacturing and processing	48.9%	43.0%
58	Architecture and building	41.8%	37.2%
62	Agriculture, forestry, and fishery	45.0%	47.2%
64	Veterinary	55.6%	79.7%
72	Health	64.5%	58.5%
76	Social services	72.6%	75.5%
81	Personal services	53.9%	46.1%
85	Environmental protection	63.7%	n.a.
86	Security services	31.5%	n.a.

^a^Data sources: Bulgarian National Statistical Institute, students enrolled in BA and MA programs, nationally pooled data for 2008–2013 (Bulgaria); Swiss Federal Statistical Office, Students and graduates in Swiss higher education institutions, pooled data for 2000–2010 (Switzerland)

**Table 12:** Classification of educational programs (ISCED97 2-digits) and share of women per educational program in Bulgaria and Switzerland: Colleges (Bulgaria)/Universities of applied sciences (Switzerland)

ISCED	Description	% of women in educational program^a^	
		Bulgaria (Colleges)	Switzerland (UAS)
14	Teacher training and education science	85.1%	62.3%
21	Arts	63.7%	54.4%
22	Humanities	n.a.	70.5%
31	Social and behavioural science	57.8%	n.a.
32	Journalism and information	n.a.	60.7%
34	Business and administration	60.1%	37.5%
44	Physical sciences	n.a.	13.5%
48	Computing	27.5%	13.4%
52	Engineering and engineering trades	28.6%	8.4%
54	Manufacturing and processing	63.4%	37.6%
58	Architecture and building	28.0%	23.6%
62	Agriculture, forestry, and fishery	32.3%	30.6%
72	Health	71.1%	86.7%
76	Social services	78.9%	74.8%
81	Personal services	54.8%	53.1%
85	Environmental protection	68.4%	44.3%

^a^Data sources: Bulgarian National Statistical Institute, students enrolled in professional BA programs, nationally pooled data for 2008–2013 (Bulgaria); Swiss Federal Statistical Office, Students and graduates in Swiss higher education institutions, pooled data for 2000–2010 (Switzerland)

### Appendix 2

See Table 13.

**Table 13:** Classification of occupational groups (ISCO88 2-digit) and share of women per occupational group in Bulgaria and Switzerland

ISCO	Description	% of women in occupational group^a^	
		Bulgaria	Switzerland
11	Legislators and senior officials	68.2%	40.9%
12	Corporate managers	39.5%	n.a.
13	Managers of small enterprises	33.2%	n.a.
21	Physical, mathematical, and engineering science professionals	31.2%	14.2%
22	Life science and health professionals	63.9%	55.0%
23	Teaching professionals	88.0%	62.2%
24	Other professionals	71.1%	38.8%
31	Physical and engineering science associate professionals	24.3%	21.1%
32	Life science and health associate professionals	85.2%	87.8%
33	Teaching associate professionals	78.4%	76.3%
34	Other associate professionals	29.6%	47.5%
41	Office clerks	66.4%	26.0%
42	Customer services clerks	80.9%	63.4%
51	Personal and protective services workers	49.2%	63.0%
52	Models, salespersons, and demonstrators	67.5%	66.9%
61	Skilled agricultural and fishery workers	35.2%	27.4%
71	Extraction and building trades workers	1.6%	2.9%
72	Metal, machinery, and related trades workers	5.1%	5.4%
73	Precision, handicraft, craft printing, and related trades workers	35.0%	40.5%
74	Other craft and related trades workers	60.6%	22.6%
81	Stationary plant and related operators	17.4%	12.1%
82	Machine operators and assemblers	58.5%	16.8%
83	Drivers and mobile plant operators	1.7%	7.3%
91	Sales and services elementary occupations	52.1%	59.9%
92	Agricultural, fishery, and related laborers	30.7%	30.8%
93	Laborers in mining, construction, manufacturing, and transport	16.5%	22.6%

^a^Data sources: Bulgarian Labour Force Survey, 15–34-year-olds, pooled data for 2006–2010 (Bulgaria); Swiss Census 2000, 15–35-year-olds, actual job (Switzerland)

### Appendix 3: Derivation of the Mutual Information Index

The Mutual Information Index is based on the concept of entropy. Entropy measures are based on the amount of additional information one gains about an outcome by knowing a particular characteristic of the individual. In the following, we refer to Technical Appendix A in DiPrete et al. (2017, 1918–1923).

In DiPrete et al., entropy reflects the expected gain in information about one’s educational program if we know his or her actual educational program. It can be measured as follows (DiPrete et al. 2017, 1919):

$$E(P_{g} ) = \sum\limits_{g = 1}^{G} {p_{g} \log \left( {\frac{1}{{p_{g} }}} \right)}$$

where *g* is the educational program and *p*_*g*_ is the probability of being in educational program *g*. The entropy reaches its minimum when everyone clusters in the same educational category and the maximum number of people are equally distributed over the educational categories.

The main gain of the Mutual Information Index (*M*) is to know by how much the entropy of educational programs is being reduced if we know an individual’s occupational category. If the entropy reduces a lot, we observe a strong linkage between education and occupation, and *M* reaches its maximum value. Following DiPrete et al., *M* “measures the average reduction in entropy in *p*_*g*_ between its overall value and its value within a specific occupation, averaged over all occupations” (ibid.):

$$M = \sum\limits_{j = 1}^{J} {p_{j} \left( {E(P_{g} ) - E(P_{g\left| j \right.} )} \right)}$$

where *j* represents the different occupational categories. If the average reduction in entropy by knowing one’s occupational category is high, the second term of the equation is much higher than the first term and *M* is high (which reflects high linkage strength).

*M* is influenced by the distribution of people across educational and occupational categories (*p*_*j*_ and *p*_*g*_). As this poses a problem for comparative analysis, following DiPrete et al. (2017, 1921f), we decompose *M* into three parts, one of which is the composition invariant (ΔN). The two other parts are ΔE and ΔO, which influence *M* only through variations in the distribution of occupational programs ΔN and educational programs ΔE. The decomposition can be written as:

$$M_{tk} - M_{{tk^{\prime}}} = \Delta N_{g} + \Delta O_{g} + \Delta E_{g}$$

where *M*_*tk*_* − M*_*tk′*_ is the difference in the Mutual Information Index between the two countries.

ΔN, ΔO, and ΔE can be calculated as follows (DiPrete et al. 1922, 2017):

$$\Delta N_{g} = \frac{1}{2}\Delta N\left( {P_{g.} (k)} \right) + \frac{1}{2}\Delta N\left( {P_{g.} (k^{\prime})} \right)$$

$$\Delta N\left( {P_{g.} (k)} \right) = \sum\limits_{g = 1}^{G} {p_{g} (k)\sum\limits_{j = 1}^{J} {\left\{ {p_{j\left| g \right.} (k)\log \left( {p_{j\left| g \right.} (k)} \right) - p_{j\left| g \right.} (k^{\prime})\log \left( {p_{j\left| g \right.} (k^{\prime})} \right)} \right\}} }$$

$$\Delta O_{g} = T_{occ} (k^{\prime}) - T_{occ} (k)$$

$$T_{occ} (k) = \sum\limits_{j = 1}^{J} {p_{j} (k)\log \left( {\frac{1}{{p_{j} (k)}}} \right)}$$

$$\Delta E_{g} = \frac{1}{2}\Delta E\left( {P_{g} (k)} \right) + \frac{1}{2}\Delta E\left( {P_{g} (k^{\prime})} \right)$$

$$\Delta E\left( {P_{g} (k)} \right) = \sum\limits_{{t = k,k^{\prime}}} {\left\{ {\sum\limits_{g = 1}^{G} {\left( {p_{g} (k^{\prime}) - p_{g} (k)} \right)} \;\sum\limits_{j = 1}^{J} {p_{j\left| g \right.} (g)\log \left( {p_{j\left| g \right.} (k)} \right)} } \right\}}$$

where *P*_*g*_*(k)* (*P*_*g*_*(k′))* is the distribution across the educational categories in country *k (k′)*, *p*_*g*_*(k)* is the share of people of country *k* who are in the educational program *g,* and *p*_*j|k*_ is the likelihood of being in occupational category *j*, given one’s educational program is *g*. See DiPrete et al. (2017) for further details.

## Footnotes

1 An additional examination has to be passed to study at a university with an FVB; few students make use of this opportunity (Grob and Leu 2007).

2 The authors explain the strong gender segregation in initial vocational education by institutional age and the connected independence and autonomy in occupational choice. Young people who need to enrol in VET have to decide on a particular training occupation at the age of 15, while students who enrol in general upper secondary schools can decide at a later point in time. It is argued that occupational choices made during adolescence are especially gender typed, because at this age gender identity and the resulting gender stereotypical choices are particularly pronounced (Buchmann and Charles 1995).

3 The age of respondents at the start of the first significant job is between 15 and 34 in Bulgaria and between 17 and 30 in Switzerland.

4 ISCED97 was the legal basis in Bulgaria in education at the time when the BSLS 2014 data was collected.

5 A weakness of the dissimilarity index is that changes in the size of educational/occupational categories alter its value, even when gender ratios remain unchanged. Despite this well-known limitation, the dissimilarity index is still considered a fairly good measure of overall gender segregation (Blackburn 2012).

6 For a more detailed derivation of the Mutual Information Index, see Appendix 3.

7 As a rough guideline on linkage strength (*M*), the share of people in the three most common occupational categories (given a specific group of educational categories) can be calculated as one-fifth of the linkage strength plus 0.2 (DiPrete et al. 2017: 1895). For example, if a linkage strength (*M*) is 0.5, the share of people with a given educational credential, clustering in the three most common occupations of this educational credential, is 0.5 * 0.2 + 0.2 = 0.3. Thus, a linkage strength of 0.5 can be interpreted as 30% of all individuals with a given educational credential clustered in the three most common occupations of that educational credential.

8 Because our dependent variable is bounded (ranging from 0 to 100%) and OLS regression models imply a potential infinite range below and above zero, we have additionally tested all models with the logit of gender concentration as dependent variable. The results reported in Sect. 6.3 remain mainly the same. Although the significance level of our findings for women in BG and CH rises to 10% and some effect sizes change slightly, the direction of the statistical effects remains unchanged.

9 The measure we use to calculate the structural linkage strength (*M*) takes into account the different distributions of educational and occupational categories per country, but not the different distribution of those categories between the countries. *M* can be broken down into a composition-invariable term that can be compared between the two countries. The decomposition, however, allows only for the identification of the composition invariable *difference* in *M* between the countries, and not for the identification of composition-invariable country-specific linkage strengths.

10 The robustness of all four OLS models presented below has been tested by additionally controlling for migrant background (for Switzerland) and ethnicity (for Bulgaria). Whereas some of those controls show significant main effects on gender concentration in employment—with male Turks as well as female Romani people in Bulgaria and first-generation women in Switzerland having more gender-typical employment—they do not change our target independent variables which are the gender concentration of the educational program and its interaction with the linkage strength.

11 In the case of Switzerland, we do not control for firm mobility or any takeover behaviour by the training company. While takeover will most probably result in the direct transmission of a gendered vocational education into a gendered job, Müller and Schweri (2015) have shown that only one in seven graduates who *change the firm* also change their learned occupation in Switzerland. Hence, firm mobility and firm takeover should result in a similar transmission of gender segregation from education to the first significant job.

12 We have not derived any assumptions about how gender may moderate the linkage strength of education and early employment in Switzerland and Bulgaria. This is because our institutional arguments developed in Sect. 3 do not allow for straightforward gender-specific propositions. Our findings are therefore of an explorative nature and need further theorizing to interpret the empirical pattern reported in this paragraph.

13 Due to the small number of observations of college graduates in Bulgaria, we do not include a country comparison between the UAS and the colleges.

14 For example, ΔE Total = 0.29 means that the difference in the raw linkage strength between Switzerland and Bulgaria of 0.62 is overestimated by 0.29 due to the different distribution of educational categories.

15 ΔE shows that women with vocational education in Switzerland have a weaker linkage of education and work than in Bulgaria. The Swiss phenomenon is however not of a structural nature as becomes clear from ΔN; it is conditioned by the fact that women in Switzerland are often disproportionately completing educational programs with a weak link (and less often educational programs with a strong link). An example of such an educational program is the commercial sector, in which many women in Switzerland obtain their degree. From research, it is known that educational training in commercial occupations conveys wider, i.e. less specific, qualifications (Hall 2010, 2011). People in this sector can work in relatively heterogeneous occupational fields, given the relatively broad skills conveyed in the commercial training.

16 Gender concentration in education is measured in deciles. As such, the marginal effects of this variable reflect an increase in the average male share in the completed educational program by 10 percentage points.

17 A robustness check in which we additionally controlled for migration experience has shown that men *without* migration background who completed a UAS are actually *increasingly* accessing male-typed jobs compared to those who directly transitioned from VET to the labour market. As the effect of a university degree fades away for the same group, the respective negative effect in Table 6 might be conditioned by graduates with migration background.

18 The reported findings are based on the international classifications of the first significant job and the educational program (ISCO88 and ISCED97), which are officially used in Bulgaria to make our analysis comparable. We additionally calculated the models for Switzerland using more fine-graded Swiss educational and occupational classifications to check for robustness. The findings remain the same for the different classification systems.

19 The interpretation of country differences regarding the linkage strength should however be approached with caution because we could not calculate a joint model for Switzerland and Bulgaria. Identification of a *composition-invariable country specific* linkage strength is not possible (see footnote 9).
